# Protective Effect of Vegan Microbiota on Liver Steatosis Is Conveyed by Dietary Fiber: Implications for Fecal Microbiota Transfer Therapy

**DOI:** 10.3390/nu15020454

**Published:** 2023-01-15

**Authors:** Nikola Daskova, Marie Heczkova, Istvan Modos, Jaromir Hradecky, Tomas Hudcovic, Marek Kuzma, Helena Pelantova, Irena Buskova, Eva Sticova, David Funda, Jaroslav Golias, Barbora Drabonova, Jarmila Jarkovska, Maria Kralova, Ivana Cibulkova, Jan Gojda, Monika Cahova

**Affiliations:** 1 Institute for Clinical and Experimental Medicine, 14021 Prague, Czech Republic; 2 First Faculty of Medicine, Charles University, Katerinska 1660/32, 12108 Prague, Czech Republic; 3 Faculty of Forestry and Wood Sciences, Czech University of Life Sciences, 16500 Prague, Czech Republic; 4 Institute of Microbiology of the CAS, 14220 Prague, Czech Republic; 5 Faculty of Agrobiology, Food, and Natural Resources, Czech University of Life Sciences, 16500 Prague, Czech Republic; 6 Department of Applied Mathematics and Computer Science, Masaryk University, 60177 Brno, Czech Republic; 7 Department of Internal Medicine, Kralovske Vinohrady University Hospital and Third Faculty of Medicine, Charles University, 10000 Prague, Czech Republic

**Keywords:** fecal microbiota transfer, vegan microbiota, liver steatosis, inulin, proteolytic fermentation

## Abstract

Fecal microbiota transfer may serve as a therapeutic tool for treating obesity and related disorders but currently, there is no consensus regarding the optimal donor characteristics. We studied how microbiota from vegan donors, who exhibit a low incidence of non-communicable diseases, impact on metabolic effects of an obesogenic diet and the potential role of dietary inulin in mediating these effects. Ex-germ-free animals were colonized with human vegan microbiota and fed a standard or Western-type diet (WD) with or without inulin supplementation. Despite the colonization with vegan microbiota, WD induced excessive weight gain, impaired glucose metabolism, insulin resistance, and liver steatosis. However, supplementation with inulin reversed steatosis and improved glucose homeostasis. In contrast, inulin did not affect WD-induced metabolic changes in non-humanized conventional mice. In vegan microbiota-colonized mice, inulin supplementation resulted in a significant change in gut microbiota composition and its metabolic performance, inducing the shift from proteolytic towards saccharolytic fermentation (decrease of sulfur-containing compounds, increase of SCFA). We found that (i) vegan microbiota alone does not protect against adverse effects of WD; and (ii) supplementation with inulin reversed steatosis and normalized glucose metabolism. This phenomenon is associated with the shift in microbiota composition and accentuation of saccharolytic fermentation at the expense of proteolytic fermentation.

## 1. Introduction

A Western-type diet characterized by a high intake of refined sugars, animal fats, and processed food, is associated with a sharp increase in the prevalence of obesity and non-alcoholic fatty liver disease (NAFLD), one of the most common liver diseases worldwide [1,2]. Gut microbiota have repeatedly been shown to be among the most important mediators between diet and obesity risk [3,4]. Several mechanisms were proposed: enhanced energy harvest, central effects on satiety perception, impairment of intestinal barrier function, and promotion of chronic inflammation [5]. Certain metagenomic patterns associated with obesity have been described in the literature [6,7], but they are significantly influenced by factors such as age, ethnicity, and geography [8].

Up to now, there is no proven pharmacological treatment for NAFLD and the therapeutic strategies are based mostly on lifestyle interventions, namely, diet [9]. There are numerous dietary regimes aimed at weight loss and the improvement of metabolic health [10,11]. Modulation of nutrient intake has several direct effects on the host physiology, such as via nutrient load. In addition, rapidly emerging research into the essential role of the human microbiome in host physiology opened up the question of whether the beneficial effects of various diets could be mediated, in some aspects, by shaping the intestinal microbiota and changing its metabolic programming. Besides delivery of prebiotic substrates or probiotic intervention, fecal microbiota transfer (FMT), a currently approved therapeutic approach in the treatment of *Clostridioides difficile* infection [12], has gained growing attention in the context of therapy of other non-communicable diseases, including obesity or metabolic syndrome [13]. In contrast to probiotic treatment, which does not induce an alteration in microbiota composition [14], FMT causes a structural change in the whole gut microbial community [12], and thus may convey complex beneficial effects. Up to now, six randomized clinical trials assessing the use of FMT from lean omnivore donors in obese and metabolic syndrome patients have been reported [13,15,16,17,18]. Meta-analysis of the data showed only a partial effect of FMT on obesity-related disorders. While there was a significant reduction in HbA1c, HDL, and LDL cholesterol levels in FMT recipients, there was no modification of weight, serum TAG content, or reduction in glycemia [19]. However, none of these studies included any dietary recommendations after FMT.

The effective use of FMT is limited by the insufficient definition of the optimal donor. According to the current literature, a vegan diet is considered a metabolic health-promoting approach [20,21,22,23,24], and diet is one of the main environmental factors modulating the composition of gut microbiota [25]. Therefore, vegans were implied as suitable donors, though with unequivocal results. It was shown that vegan FMT alone had a modestly beneficial effect in the treatment of steatohepatitis [26] but failed to elicit changes in trimethylamine-N-oxide production in patients with metabolic syndrome [17]. As the receivers did not change their eating habits, the vegan microbiota itself were probably unable to counteract the effect of the unhealthy obesogenic diet. In line with this observation, our previous study showed that strong adherence to a vegan diet in humans resulted in only a relatively mild effect on microbiota composition [27]. On the other hand, veganism was associated with significant modification of microbiota performance towards the beneficial metabolite spectrum, but this aspect could be manifested only in combination with a diet rich in plant-based food.

Despite these preliminary data, it has not been explored how the transferred microbiota are affected by the host gut environment and the substrates provided. Therefore, we aimed to seek whether the metabolic effects of vegan-derived FMT are mediated by diet, and particularly by dietary fiber. Using the model of ex-germ-free mice colonized with human vegan microbiota, we explored (i) how vegan microbiota protects against obesogenic (Western-type) diets; (ii) mechanistic relations in microbiome/metabolome composition, and (iii) the ability of dietary fiber (inulin) to enforce vegan microbiota therapeutic potential in the obesogenic milieu.

## 2. Materials and Methods

### 2.1. Gut Microbiota Donors

Four vegan donors who did not object to animal experiments for ethical reasons were recruited from the vegan cohort described in detail in our previous study [27]. Their clinical characteristics are given in Table 1 and the Supplemental File S1.

All of them strictly avoided all animal products for at least three years. The exclusion criteria were chronic diseases related to metabolism, diseases of the digestive tract, antibiotic therapy in the past three months, pregnancy, any chronic medication (excluding hormonal contraception), and regular alcohol consumption defined as any alcoholic drink on a daily basis. The participants were asked to donate a fresh stool sample, which was immediately processed [28]. The total bacteria number in each sample was assessed by quantitative pan-bacterial real-time PCR (forward primer ACACTGACGACATGGTTCTACAGAGTTGATCNTGGCTCAG, reverse primer TACGGTAGCAGAGACTTGGTCTGTNTTANGCGGCKGCTG) and each inoculum was diluted to approx. the same bacterial abundance (3.9 × 10^6^ CFU/μL). The mixed VG inoculum was prepared by mixing an equal amount of each sample. The aliquots were kept frozen with buffered glycerol at −80 °C until the transfer.

### 2.2. Animals

**Figure 1:**
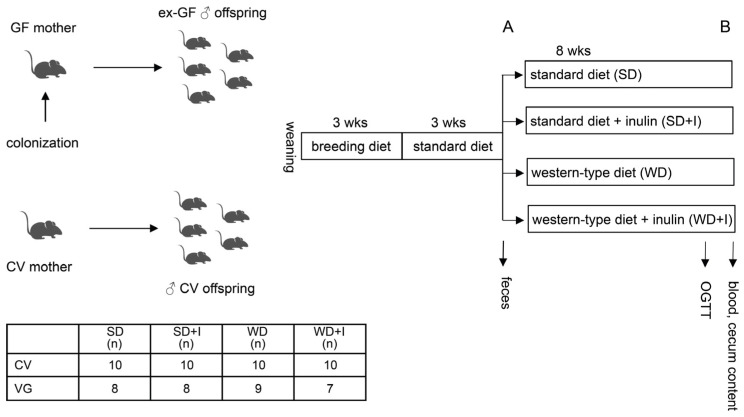
Experimental design. CV, conventional mice; GF, germ-free; OGTT, oral glucose tolerance test. (**A**), timepoint A (prior diet intervention); (**B**), timepoint B (after diet intervention).

Germ-free C57Bl6 mice originated from the colony bred at the Gnotobiology laboratory Institute of Microbiology of the CAS, Novy Hradek, CR. Mice were kept under sterile conditions in Trexler-type plastic isolators, exposed to 12:12 h light–dark cycles, and supplied with autoclaved tap water and 50 kGy irradiated sterile pellet (breeding diet: Altromin 1414, Altromin, Germany) ad libitum. Axenicity was assessed every two weeks by confirming the absence of bacteria, mold, and yeast by aerobic and anaerobic cultivation of mouse feces and swabs. Female mice were colonized by mixed VG inoculum, bacterial load ≥1 × 10^9^ bacteria by the means of administration on the skin, enema, and oral gavage. The colonized females were mated to germ-free males. Their male offspring, further described as VG, were kept in gnotobiotic isolators and used for the experiments. We decided to adopt this design because we aimed to create a “physiologically normal” mouse model colonized with human vegan microbiota. Recent evidence shows that maternal exposure to intestinal microbes triggers a wide range of adaptations in the offspring and the pups born to colonized mothers differ from germ-free mice colonized later during their lifetime [29,30]. Another reason is that with maternal colonization, we achieve high homogeneity of offspring colonization. After weaning, all animals were fed a breeding diet for 3 weeks. Conventional C57Bl6 mice (CV) were obtained from the breeding facility of the Institute of Microbiology of the CAS, Prague, CR. The power analysis was calculated to estimate the minimal number of animals per group according to the main outcome variable, liver TAG content (min n = 5 for ***p*** ˂ 0.05 with 0.8 probability). At time point A, mice were randomly divided into four groups, each of them receiving a specific diet for another 8 weeks: SD (standard diet), SD + I (standard diet + 10% inulin), WD (western diet), WD + I (western diet + 10% inulin). The experimental design is shown in Figure 1. After 8 weeks (timepoint B), an oral glucose tolerance test was performed, animals were killed by an overdose of anesthesia, and tissue samples were collected for further analyses. Feces were collected at time points A and B. Experimental diets, the Western diet (42 kJ% fat, 43 kJ% carbohydrates, 15 kJ% protein, no. TD88137 mod.), and standard diet control of the Western (13 kJ% fat, 69 kJ% carbohydrates, 15 kJ% protein, no. CD88137) were bought from Ssniff (Soest, Germany). Inulin-supplemented diets (10% *wt*/*wt*) were custom-made by Ssniff. The diets were sterilized by irradiation. All animal experiments were conducted in concordance with the Guide for the Care and Use of Laboratory Animals (2011).

### 2.3. Oral Glucose Tolerance Test

An oral glucose tolerance test (OGTT) and a parallel assay of C-peptide concentration in serum were performed after the administration of a dose of glucose (1 mg. g^−1^ body weight) into overnight fasting mice. Blood was taken from the tail vein into heparinized capillaries. The blood glucose was determined with a glucometer (Roche, Basel, Switzerland) and C-peptide concentration with an ELISA kit (Mercodia, Uppsala, Sweden). Sampling was performed at 0, 30, 60, and 120 min for each mouse.

### 2.4. Stool and Cecum Content Bacteriome Analysis

Stool samples were collected from each mouse individually at timepoint A, and the cecum content was collected at timepoint B. Samples were kept at −80 °C until the DNA was extracted using a QIAmp PowerFecal DNA Kit (Qiagen, Hilden, Germany), and the V4 region of the bacterial 16S rRNA gene was amplified by PCR. Sequencing was performed with the MiSeq reagent kit v2 using a MiSeq instrument (Illumina, Hayward, CA, USA). Raw sequences were processed using a DADA2 amplicon denoiser [31]. Subsequent taxonomic assignment was conducted by the assignTaxonomy function from the DADA2 R package using the Silva 138.1 reference database [32] at the levels L_1 (Phylum), L_2 (Class), L_3 (Order), L_4 (Family), L_5 (Genus) and L_6 (Species).

### 2.5. Volatile Compounds (VOCs) Analysis in Feces

Cecum content was homogenized and diluted to the equivalent of 1% (*wt*/*wt*) dry mass. Volatile fingerprinting of fecal samples was performed using an Agilent 7890B gas chromatograph (Agilent Technologies, Canta Clara, CA, USA) coupled to a Pegasus 4D time of flight mass spectrometer (LECO, USA). Volatiles were collected using solid-phase microextraction (SPME) fiber with a divinylbenzene/carboxen/polydimethylsiloxane coating from Supelco (USA). Data acquisition and initial data processing were carried out using instrumental SW ChromaTOF by LECO.

### 2.6. NMR Analyses

Serum samples (after protein precipitation) were measured on a 600 MHz Bruker Avance III spectrometer (Bruker BioSpin, Rheinstetten, Germany) equipped with a 5 mm TCI cryogenic probe head. 1D-NOESY, CPMG, and *J*-resolved experiments were performed using standard manufacturers’ software Topspin 3.5. The concentrations of individual metabolites, identified by comparison of proton and carbon chemical shift with the HMDB database, were expressed as PQN [33] normalized intensities of corresponding signals in CPMG spectra. The list of quantified metabolites with corresponding ^1^H and ^13^C chemical shifts is given in the Supplemental Methods.

### 2.7. Triglyceride (TAG) Content in the Liver

Lipids were extracted from 100 mg of fresh liver tissue homogenized in 2 mL of 5%NP40 in deionized H_2_O (95 °C, 5 min; room temperature, 10 min; 95 °C, 5 min). The mixture was vortexed between the steps. After the extraction, 50 μL was used for the determination of protein concentration, and the rest of the homogenate was centrifuged for 3 min at 14,000× *g*. The clear supernatant was diluted 1:9 in deionized H_2_O. The triglyceride concentration was determined using a commercially available kit (ERBA-Lachema Diagnostics, Czech Republic) and expressed as μmol TAG mg prot^−1^.

### 2.8. Statistics

The statistical analyses were performed using R software packages and in-house scripts [34]. Prior to all statistical analysis, stool and cecum taxa were filtered. We kept only taxa that appeared in at least 5% of the samples. Stool and cecum microbiota univariable analysis was performed with the DESeq2 R package [35] on raw read counts. When more than one group is compared, we report both the *p*-values from the likelihood ratio test and the pairwise Wald tests. The *p*-values were adjusted using the Benjamin–Hochberg correction. The reported effect size for stool and cecum microbiota is a log 2-fold change obtained by DESeq2. The significance in univariable analysis for NMR and VOC data was obtained by the Kruskal–Wallis test and Dunn’s post hoc test with the Benjamin–Hochberg correction. The reported effect size for these datasets is Cliff’s delta. Alpha diversity in stool and cecum microbiota was analyzed with a vegan R package (https://CRAN.R-project.org/package=vegan, accessed on 16 November 2022). The raw counts were rarefied to 10,000 reads and then the Shannon index was computed. Statistical significance was computed by the Kruskal–Wallis test and Dunn’s post hoc test with Benjamin–Hochberg correction. Principal component analysis (PCA) and Permutational MANOVA (PERMANOVA, *adonis* function from R package vegan) was used for multivariable analysis. All the datasets were centered and scaled prior to analysis. Additionally, prior to further analysis stool and cecum datasets were transformed by Variance Stabilizing Transformation. For assessing statistical significance, PERMANOVA was run with 10,000 iterations. We used Euclidean distance in both PCA and PERMANOVA. For pairwise PERMANOVA comparison, R package pairwiseAdonis was employed. To determine the discriminating ability of each dataset, we created classification models using Lasso. The classification metrics were obtained using 3-fold cross-validation with 10 repeats. To select the smallest number of variables in the models, we reported lambda = 1 se, that is, a lambda that results in the most regularized model so that the cross-validated error is within one standard error of the minimum error. Correlation networks of liver TAG content with microbiome were created as follows. First, the significantly changed taxa from the univariable analysis were identified. The Spearman correlation coefficient was computed on these taxa against the liver TAG content. The *p*-values corresponding to the correlation coefficients were adjusted using the Benjamin–Hochberg correction and the correlation networks were created with correlations having adjusted *p*-value ≤ 0.1. For the stool dataset on L_5 and L_6 levels, we show only correlations with an absolute value larger than 0.6.

## 3. Results

### 3.1. Body Composition and Glucose Homeostasis

**Figure 2:**
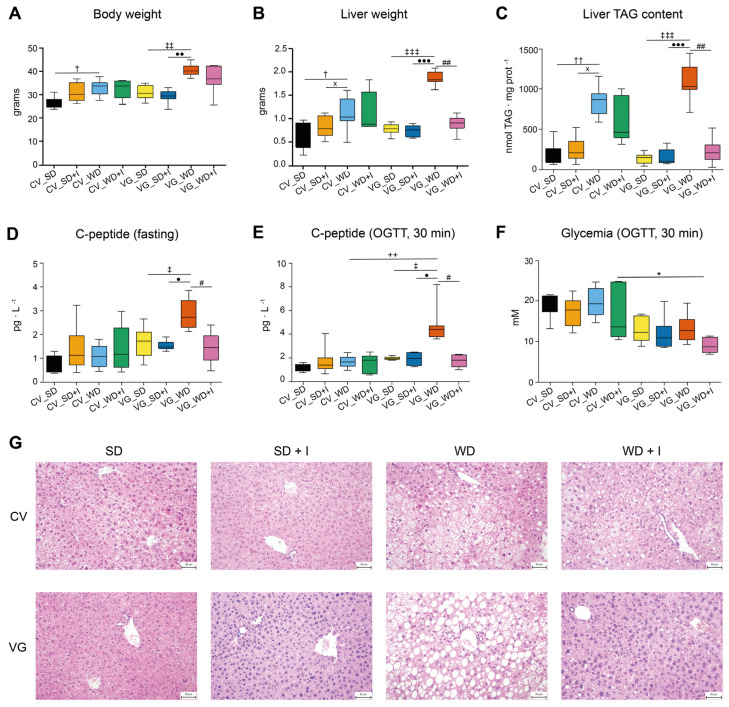
Effect of the diet and inulin supplementation on body composition and glucose homeostasis. (**A**): total body weight (g); (**B**): liver weight (g); (**C**): liver TAG content (nmol TAG. mg^−1^ protein); (**D**): fasting C-peptide concentration in plasma (pg. L^−1^); (**E**): C-peptide concentration in plasma at 30 min of OGTT (pg. L^−1^); (**F**): glycemia at 30 min of OGTT (mM); (**G**): histological assessment of liver slices. Data are shown as box plots (first and third quartile, median) with whiskers (min, max). OGTT, oral glucose tolerance test; CV, conventional mice; VG, humanized mice; SD, standard diet; SD + I, standard diet supplemented with inulin; WD, Western diet; WD + I, western diet supplemented with inulin. ^†^ *p* ˂ 0.05, ^††^ *p* ˂ 0.01 CV_WD vs CV_SD; ^x^ *p* ˂ 0.05 CV_WD vs CV_SD + I; ^‡^ *p* ˂ 0.05, ^‡‡^ *p* ˂ 0.01, ^‡‡‡^ *p* ˂ 0.001 VG_WD vs VG_SD; ^•^ *p* ˂ 0.05, ^••^ *p* ˂ 0.01, ^•••^ *p* ˂ 0.001 VG_WD vs VG_SD + I; ^#^ *p* ˂ 0.05, ^##^ *p* ˂ 0.01 VG_WD + I vs VG_WD; ^++^ *p* ˂ 0.01 VG_WD vs CV_WD; ٭ *p* ˂ 0.05 VG_WD + I vs CV_WD + I.

We assessed the effect of the experimental diets on phenotype according to body composition parameters, that is, total body weight, liver weight, and triacylglycerol (TAG) content in the liver in ex-germ-free humanized mice (VG) and conventional mice (CV) (Figure 2).

As expected, the western diet induced a significant increase in total body and liver weight and liver TAG content in both models, the latter two parameters being significantly more affected in VG compared with CV mice. Inulin supplementation had no effect on WD-induced changes in CV mice, but it was associated with the decrease of liver TAG content and liver weight reaching a normal level in VG mice. We also observed a tendency to the normalization of epididymal fat pad weight in the VG_WD + I group (not shown), despite it not reaching statistical significance at *p* ˂ 0.05. Western diet administration was associated with the deterioration of insulin secretion assessed as the fasting and glucose-stimulated C-peptide serum concentration only in VG mice. The effect of WD was compensated by inulin treatment, as both parameters were normalized in the VG_WD + I group. Rather surprisingly, the glycemia at 30 min OGTT tended to be higher in CV mice, and in VG_WD + I it was even significantly lower compared with the CV_WD + I group. Because of the lack of the effect of inulin on metabolic phenotype in CV mice, we further focused on the VG model aiming to identify the components of the microbiome and metabolome associated with the beneficial outcome.

### 3.2. Cecum Microbiota Composition after Dietary and Inulin Intervention in Humanized Mice

**Figure 3:**
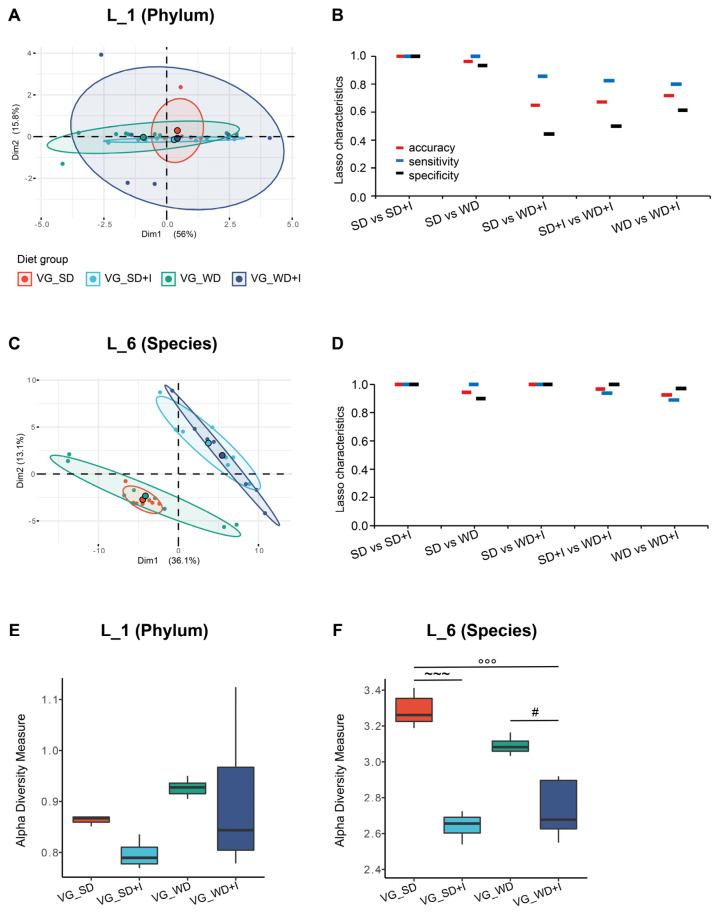
Cecum microbiota composition in humanized mice. (**A**,**C**): The 2D PCA scores plot. The explained variance of each component is included in the axis labels. The large points represent the centroids of each group. (**B**,**D**): Held-out characteristics of Lasso logistic regression model. (**E**,**F**): Alpha diversity of cecum microbiota assessed as Shannon index. ~~~ *p* ˂ 0.001 VG_SD + I vs VG_SD; °°° *p* ˂ 0.001 VG_WD + I vs VG_SD; ^#^ *p* ˂ 0.05, *p* VG_WD + I vs VG_WD. Data are shown as box plots (1st and 3rd quartile, median) with whiskers (min, max).

Prior to the interventions (timepoint A), the microbiome diversity and composition in feces did not differ in mice randomly allocated to experimental groups (Figures S1 and S2). Aiming to compare the microbiota composition among the experimental groups after the intervention (timepoint B), we analyzed the cecum content because we consider it the most representative sample of microbiota in the distal intestine. The unsupervised separation of groups by variance only was visualized using PCA (Figure 3A,C). At the phylum level, the composition of the microbiota did not differ, PERMANOVA *p* = 0.025 but the analysis of dispersion test was significant which influence the result. At the species level, PERMANOVA *p* ˂ 0.001, analysis of dispersion test was insignificant and pairwise tests proved differences between all groups (SD vs SD + I *p* = 0.0011, SD vs WD *p* = 0.0011, SD vs WD + I *p* = 0.0011, WD vs WD + I *p* = 0.0042).

As the next step, we adopted a machine learning approach (Lasso logistic regression) allowing for quantification of discrimination between every two pairs of groups (Figure 3B,D). At the L_1 level, we were able to reliably discriminate only VG_SD + I vs VG_SD and VG_WD vs. VG_SD groups, but the discrimination between other groups was unsatisfactory. The precision of separation increased along with reaching lower taxonomical levels. At the L_6 level, all pairs of groups could be separated with at least 90% accuracy, sensitivity, and specificity. Alpha diversity was assessed according to the Shannon index. Western diet alone increased the diversity at the L_1 (phylum) level but did not affect other taxonomical levels. Inulin treatment negatively affected the diversity at all levels independently of the background diet (VG_SD or VG_WD) (Figure 3E,F and Figure S3).

The particular bacterial taxa discriminating between the groups were identified by univariable analysis that was performed using DESeq2. We observed an effect of all manipulations on microbiota composition at all taxonomical levels. At the L_1 level, the VG_WD group was characterized by a higher abundance of Bacteriodota, Actinobacteriota, and Verrucomicrobiota and decreased abundance of Firmicutes compared with VG_SD. Inulin treatment counteracted the WD effect on Firmicutes and potentiated the increase of Verrucomicrobia. Furthermore, inulin supplementation resulted in the decrease of Desulfobacterotota both in SD- and WD-fed mice (Table S1).

**Figure 4:**
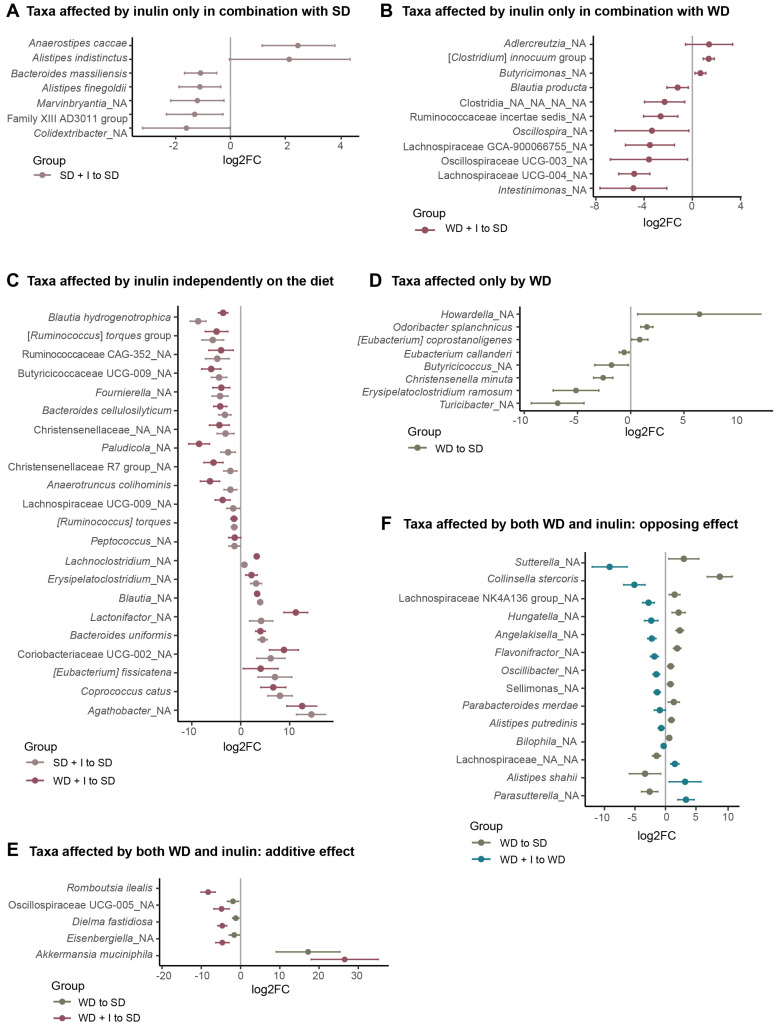
Bacterial taxa significantly affected by diet and/or inulin. (**A**): Taxa affected by inulin only in combination with SD (adj_pval VG_SD + I vs VG_SD ˂ 0.1); (**B**): Taxa affected by inulin only in combination with WD (adj_pval VG_WD + I vs VG_SD ˂ 0.1); (**C**): Taxa affected by inulin independently on the diet (adj_pval VG_SD + I vs VG_SD ˂ 0.1 and adj_pval VG_WD + I vs VG_SD ˂ 0.1); (**D**): Taxa affected only by WD (adj_pval VG_WD vs VG_SD ˂ 0.1); (**E**) Taxa affected by both WD and inulin: additive effect (adj_pval VG_WD vs VG_SD ˂ 0.1 and adj_pval VG_WD + I vs VG_WD ˂ 0.1, effect size VG_WD vs VG_SD and VG_WD vs VG_WD + I in the same direction); (**F**) Taxa affected by both WD and inulin: opposing effect (adj_pval VG_WD vs VG_SD ˂ 0.1 and adj_pval VG_WD + I vs VG_WD ˂ 0.1, effect size VG_WD vs VG_SD and VG_WD vs VG_WD + I in the opposite direction). The taxa were selected according to the outcome of the univariable statistic test (Kruskal–Wallis), omnibus adj_pval ˂ 0.1. The graph shows the effect size calculated as log2FC. adj_pval, adjusted *p*-value; FC, fold change.

At L_6 level (species), univariable analysis unraveled 76 taxonomical units, that is, 64% of all significantly differently abundant ones among groups at FDR ˂ 0.1 (Table S2). Considering the effect of diets and inulin, these bacteria could be divided into several groups (Figure 4). Forty bacterial taxa were affected only by inulin. In 18 bacteria, the effect of inulin was diet-dependent. Seven bacteria were significantly stimulated by inulin only in combination with SD (Figure 4A), and 11 only in combination with WD (Figure 4B). Twenty-two bacteria were affected by inulin independently of the background diet. Of those, inulin treatment stimulated the abundance of nine bacteria while the abundance of 13 bacteria was decreased (Figure 4C). Eight species were affected either positively (n = 3) or negatively (n = 5) only by WD (Figure 4D). In five taxa, the effect of WD was potentiated by inulin. The only bacteria whose abundance was positively affected by both WD and inulin was Akkermansia muciniphila, the effect of inulin being an order of magnitude stronger than the effect of diet. Four bacteria were affected negatively (Figure 4E). Finally, in 14 bacteria inulin counteracted the effect of WD. Eleven bacteria (Alistipes putredinis, Parabacteroides merdae, Sellimonas sp., Collinsella stercosis, Suterella sp., Hungatella sp., Flavonifractor sp., Lachnospiraceae NK4A136 group, Angelakisella sp., Oscillibacter sp., Bilophila sp.) were stimulated by WD while inulin supplementation negatively affected their abundance. Three bacteria (Parasutterella sp., Alistipes shahii, Lachnospiraceae_NA_NA) were suppressed by WD, while inulin partially compensated for this effect (Figure 4F). For nine taxa, we did not identify any pattern of diet or inulin effect.

### 3.3. Cecum VOCs Composition after the Dietary and Inulin Interventions in Humanized Mice

**Figure 5:**
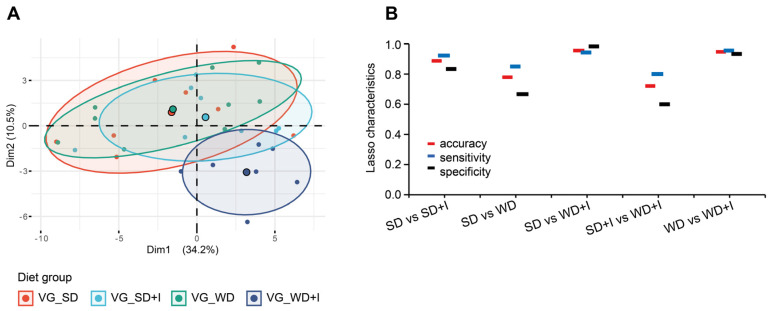
Cecum VOCs composition. (**A**) The 2D PCA scores plot. The explained variance of each component is included in the axis labels. The large points represent the centroids of each group. (**B**) Held-out characteristics of Lasso logistic regression model.

In total, we identified 61 VOCs in cecum content, 17 of them being significantly different among groups (Table S3). The separation of the groups is shown in Figure 5 and the PERMANOVA test confirmed that the VOCs composition of at least some of the groups differs. Inulin supplementation of SD did not result in a significant shift of cecum volatilome (pairwise PERMANNOVA *p* ˃ 0.1), but using the Lasso logistic regression model we were able to discriminate between VG_SD and VG_SD + I groups with 89% accuracy and 86% specificity.

**Figure 6:**
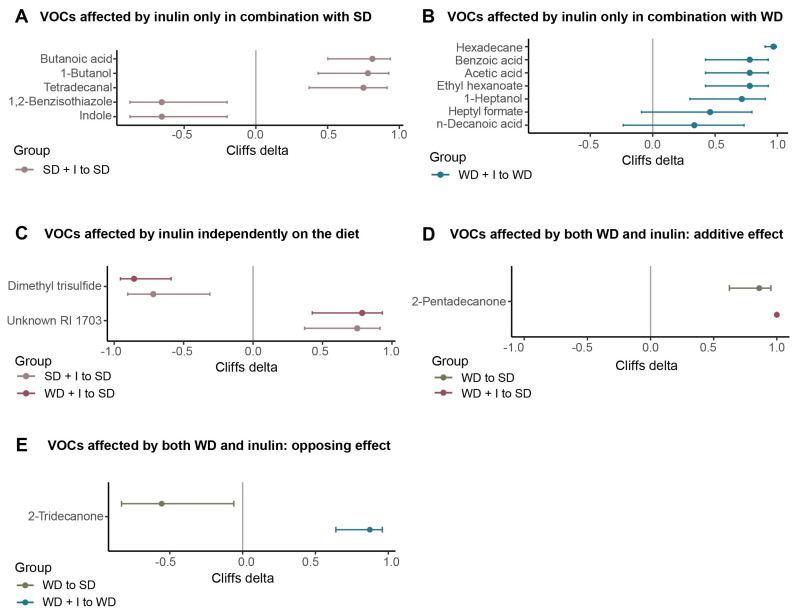
VOCs significantly affected by diet and/or inulin. (**A**): VOCs affected by inulin only in combination with SD (adj_pval VG_SD + I vs VG_SD ˂ 0.1); (**B**): VOCs affected by inulin only in combination with WD (adj_pval VG_WD + I vs VG_SD ˂ 0.1); (**C**): VOCs affected by inulin independently on the diet (adj_pval VG_SD + I vs VG_SD ˂ 0.1 and adj_pval VG_WD + I vs VG_SD ˂ 0.1); (**D**): VOCs affected by both WD and inulin: additive effect (adj_pval VG_WD vs VG_SD ˂ 0.1 and adj_pval VG_WD + I vs VG_WD ˂ 0.1, effect size VG_WD vs VG_SD and VG_WD vs VG_WD + I in the same direction); (**E**): VOCs affected by both WD and inulin: opposing effect (adj_pval VG_WD vs VG_SD ˂ 0.1 and adj_pval VG_WD + I vs VG_WD ˂ 0.1, effect size VG_WD vs VG_SD and VG_WD vs VG_WD + I in the opposite direction). The VOCs were selected according to the outcome of the univariable statistic test (Kruskal-Wallis), omnibus adj_pval ˂ 0.1. The graph shows the effect size calculated as log2FC. adj_pval, adjusted *p*-value; FC, fold change; RI, retention index.

The univariable analysis identified five compounds significantly different between both groups; indole and 1,2-benzisothiazole were significantly decreased while tetradecanal, 1-butanol, and butanoic acid were increased in VG_SD + I compared with the VG_SD group (Figure 6A). The effect of WD alone on the VOCs spectrum in the cecum was quite modest. The PERMANOVA pairwise test was non-significant (*p* = 0.374), the accuracy of the Lasso logistic regression model was 0.78 with a specificity of 0.67 and we did not identify any differences in metabolite concentrations by UDAA. The combination of WD with inulin supplementation led to the profound shift in cecum VOCs spectrum as revealed by PCA (Figure 5A), pairwise PERMANOVA result (*p* = 0.005), and the held-out characteristics of the Lasso logistic regression model (Figure 5B). Seven compounds were affected by inulin only in combination with WD (Figure 6B), all of them positive. Two compounds, unknown RI 1703 (increased) and dimethyl trisulfide (decreased), were influenced by inulin supplementation in combination with both diets (Figure 6C). Cecum content of 2-pentadecanone was increased by both WD and inulin supplementation, the effect being additive (Figure 6D). Only in the case of 2-tridecanone was the effect of inulin and WD the opposite (Figure 6E).

### 3.4. Serum Metabolome Composition after the Dietary and Inulin Interventions in Humanized Mice

The serum metabolome assessed by NMR spectroscopy was not significantly influenced by any of the treatments. PCA did not reveal any difference among the groups (all pairwise PERMANOVA tests ˃ 0.15) and the discrimination between groups based on the Lasso logistic regression model was unsatisfactory as well (Figure S4). Using univariable analysis, we did not identify any metabolite significantly different among the groups.

### 3.5. Integrative Analysis

**Figure 7:**
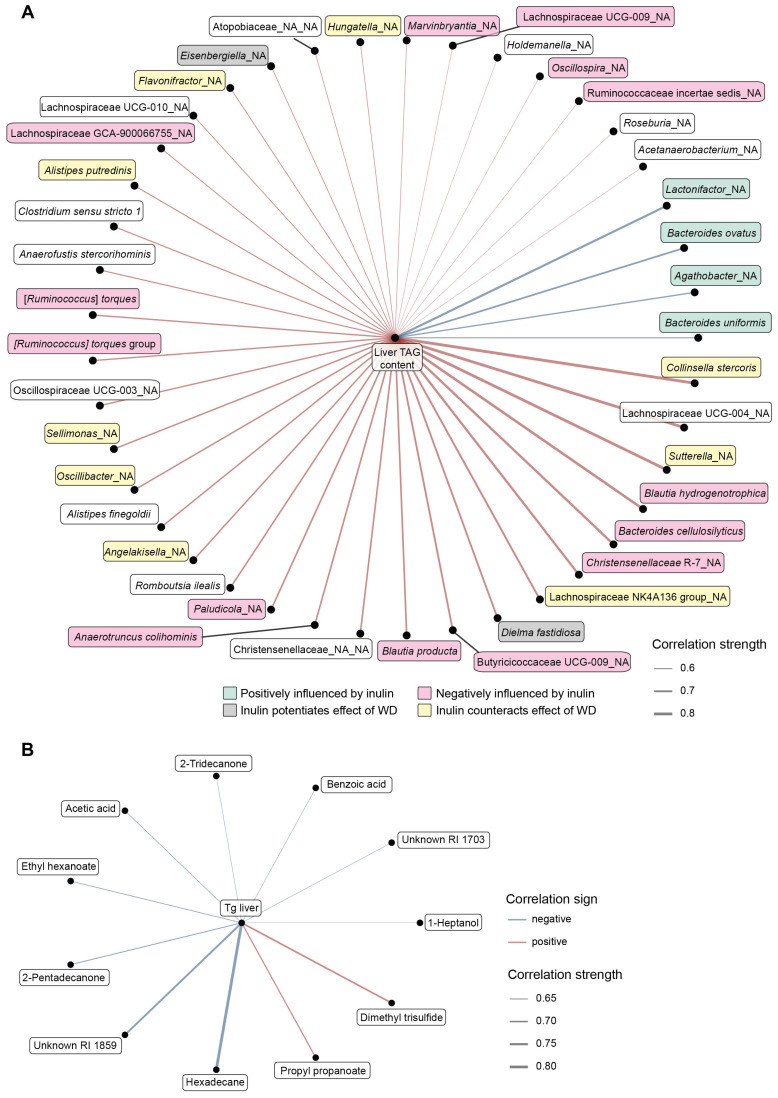
Correlation network between liver TAG content and cecum microbiome (**A**) and VOCs (**B**) composition. The edge width and color are proportional to the value of the correlation (red: positive; blue: negative). RI, retention index.

Finally, we looked for possible relationships between microbiome or VOCs composition and the attenuation of WD-induced liver steatosis. To this end, we constructed the networks based on the Spearman correlations between liver TAG content and cecum bacteria or VOCs abundance in VG_WD and VG_WD + I groups (Figure 7). Looking at the relationships between cecum microbiota and liver TAG content, we found that liver steatosis negatively correlated with the abundance of four bacteria (*Agathobacter*, *Lactonifractor*, *Bacteroides ovatus*, *Bacteroides uniformis*) that all were positively stimulated by inulin. Thirty-seven taxa correlated positively with liver TAG content including nine bacteria, whose abundance was positively affected by WD and negatively by inulin, and 12 bacteria that are negatively modulated by inulin (Figure 7A). The correlation network between liver TAG content and VOCs compounds identified in the cecum is shown in Figure 7B. We identified only two positive correlations, between liver TAG content and dimethyl trisulfide or propyl propanoate. Liver TAG content correlated negatively with nine compounds, including acetic acid, which is a marker of dietary fiber fermentation.

## 4. Discussion

We demonstrated in an animal model that vegan microbiota per se does not counteract the metabolically detrimental effects of a Western-type diet, but it shows the capacity to protect from NAFLD and glycemic deterioration when further supplemented with prebiotic inulin. The effect of inulin was manifested only in combination with vegan microbiota in humanized mice but not with conventional mice microbiota. Inulin supplementation in humanized mice resulted in a significant change in cecum microbiota composition with the accentuation of saccharolytic fermentation at the expense of the proteolytic one.

### 4.1. The Diet–Microbiota Interaction Influences the Outcomes of FMT Therapy

The landmark studies of Backhed’s group [3,4] showed that in the mice model, the obese phenotype is transmissible by gut microbiota transfer. The possibility to transfer “diseased microbiota” opens the question of whether it is possible to transfer “healthy microbiota” and to use it for therapeutic purposes. The major drawback limiting the wide application of this concept is the lack of a definition of what healthy microbiota is. In the human gut, it is not possible to define the universally applicable composition of a healthy microbiome [36]. In relation to metabolic health, adherence to plant-based diets (vegetarian or vegan) was shown to be associated with potential health benefits [37], and therefore, vegan microbiota could be considered beneficial. On the other hand, in Western countries veganism has only a modest impact on gut microbiota composition [27,38,39] and the outcomes of two available vegan FMT trials were quite modest. The potential explanation may be derived from the experiment performed by Ridaura et al. who demonstrated that the invasion and colonization potential of transferred microbiota strongly depends on the diet [40]. Germ-free mice colonized with gut microbiota from a discordant twin pair, obese (Ob) and lean (Ln), repeated the donor phenotype on a standard mice diet. When co-housed and fed a low saturated fat/high fruit and vegetables diet, Ln microbiota became dominant, invaded the gut of Ob microbiota-colonized cage mates, and prevented the development of obesity. In contrast, the dominant and protective effect of Ln microbiota disappeared when mice were fed a high saturated fat/low fruit and vegetable diet. This study demonstrates how an obesogenic diet can select against human gut bacterial taxa associated with leanness [40].

Similarly, the background microbiota determines the therapeutic effect of dietary intervention. In mice models, inulin supplementation was associated with variable outcomes. Three studies were performed on C57Bl6 mice. In one study, inulin reduced the weight gain and steatosis induced by a western diet with fructose [41] while in the other inulin did not reverse the adverse effects of a high-fat diet [42]. In mice fed an n-3 fatty acid-deficient diet, inulin treatment promoted weight gain and adiposity and did not reverse the impairment of glucose homeostasis [43]. In APOE*3-Leiden.CETP mice (atherosclerosis model) inulin did not reduce hypercholesterolemia or atherosclerosis development and even resulted in manifestations of hepatic inflammation when combined with a high percentage of dietary cholesterol [44]. In our hands, inulin supplementation did not reverse the effect of the Western diet in conventionally raised C57Bl6 mice at all. Mice intestinal microbiota is profoundly different from the human and even the mice of the same strain but from different breeding facilities substantially differ in microbiota composition. Therefore, the variable outcomes of the above-mentioned studies may result from different pre-intervention gut microbiome settings. Taken together, this evidence emphasizes the strong microbiota-by-diet interactions and the implication of this relationship for therapeutic purposes.

In the previously cited vegan FMT studies, the participants were explicitly asked not to change their habitual diet. Therefore, the modest effect of vegan donor microbiota may be attributed to the diet of the FMT receivers, which did not allow for the manifestation of FMT therapeutic potential. This hypothesis is corroborated by our previous observational study comparing the gut microbiome and metabolome of adult vegan and omnivore human cohorts [27]. We found only modest differences in fecal microbiota composition but a substantial difference in the fecal metabolome, which reflects the profoundly different diets of both groups.

### 4.2. The Protective Effect of Vegan Microbiota against Diet-Induced Steatosis Depends on Fiber Supplementation

We failed to show any protective potential of vegan microbiota to counteract the western diet-induced weight gain, namely the expansion of visceral fat. In contrast, inulin supplementation prevented the excessive TAG accumulation in the liver and ameliorated the impairment of glucose metabolism. We may suggest potential mechanisms based on our observations. Inulin supplementation induced a massive shift in microbiota composition and often counteracted the effect of an obesogenic diet. At the phylum level, inulin stimulated the abundance of Firmicutes, which resulted in the decreased Bacteroidota/Firmicutes ratio. In the presence of inulin, the abundance of Desulfobacterota was decreased both in VG_SD + I and VG_WD + I groups, which indicates the lower presence of sulfur compounds metabolizing bacteria and attenuation of potentially toxic sulfur-containing metabolites formation.

We further looked for the association between liver TAG content and the abundance of bacteria significantly affected by inulin supplementation. We found only four negative correlations between bacteria abundance and liver TAG content. All of them (*Lactonifactor* sp., *Agathobacter* sp., *B. ovatus*, *B. uniformis*) belonged to bacteria significantly stimulated by inulin. *Lactonifactor* (ρ = −0.8) converts the plant lignan secoisolariciresinol diglucoside into the bioactive enterolignans enterodiol and enterolactone [45] that have therapeutic properties, including anti-oxidant, anti-cancerous, anti-inflammatory, modulation of gene expression, anti-diabetic, estrogenic and anti-estrogenic [46]. In the cross-sectional study performed on 2294 US adults, urinary enterolactone concentration was negatively correlated with NAFLD [47]. *Agathobacter* sp. (ρ = −0.7), the bacteria most stimulated by inulin in our study, is a butyrate producer. It is reported to be stimulated by a different source of fiber (oatmeal, rye) and associated with lower cardiovascular disease or metabolic risk [48,49]. The depletion of *B. uniformis* was found in NAFLD [50,51] in observational studies. Treatment with *B. uniformis*, particularly when combined with fiber, ameliorated diet-induced hepatic steatosis and inflammation, restored the compromised intestinal immune defense, and improved whole-body glucose disposal [52,53]. Qiao et al. proposed the mechanism of the beneficial effect of *B. uniformis.* They proved that *B. uniformis* is able to synthesize folate and its beneficial effect may be explained, at least partly, by folate-enhanced one-carbon metabolism [54]. Most correlations between bacteria and liver TAG content were positive. Of interest, 38% of bacteria (n = 14) positively correlating with liver TAG is negatively influenced by inulin and in some bacteria (24%, n = 9), inulin even counteracts the effect of WD. Taken together, this evidence strongly suggests that the alteration of gut microbiota composition resulting from inulin supplementation may be responsible for the amelioration of steatosis.

### 4.3. Inulin Supplementation and Microbiota Performance

Analysis of the fecal VOCs spectrum confirmed that inulin supplementation affected the metabolic performance of cecum microbiota by accentuating saccharolytic fermentation at the expense of amino acid metabolism. This shift is documented by the (i) decrease of the product of tryptophan fermentation indole (only VG_SD + I); (ii) decrease of methionine/cysteine fermentation product dimethyl trisulfide (both VG_SD + I and VG_WD + I); (iii) increase of butanoic acid (only VG_SD + I) and (iv) increase of acetic acid (both VG_SD + I and VG_WD + I). The main fiber fermentation products are short-chain fatty acids (SCFA) whose positive effect is widely accepted [55]. Propionate and butyrate are considered unequivocally beneficial. The role of acetate is not so straightforward, as it is a lipogenic substrate and may serve as a substrate for lipid synthesis in the liver [56]. In contrast to this, Aoki et al. proposed an alternative hypothesis that acetate derived from prebiotic fermentation in the gut lumen regulates hepatic lipid metabolism and insulin sensitivity via FFAR2 signaling in hepatocytes, which prevents the progression of NAFLD [56].

The health benefits of dietary fiber have been judged mainly according to the enhanced production of SCFA in the colon. Somewhat neglected is a complementary hypothesis—the beneficial effect of dietary fiber may be mediated by the inhibition of protein fermentation as well [57]. Protein fermentation yields intrinsically toxic luminal compounds that affect epithelial cell metabolism and barrier function [58]. De Preter [57] demonstrated that oligofructose-enriched inulin in a dose-dependent fashion stimulated SCFA production and in parallel inhibited the formation of sulfur-containing compounds like dimethyl tri(di)sulfide or methional. Our data support the hypothesis that inulin supplementation attenuated the proteolytic fermentation in the colon. We observed a significantly decreased content of dimethyl trisulfide in both the VG_SD + I and VG_WD + I group and this compound strongly positively correlated with liver TAG content (ρ = 0.75). The acetic acid concentration in the cecum was significantly increased in the VG_WD + I group and negatively correlated with steatosis (ρ = −0.65). Therefore, we hypothesize that attenuation of amino acid fermentation resulting from acidification of the cecal/colonic lumen by SCFA and catabolic repression imposed by the increased saccharolytic fermentation [57] may represent an additional protective mechanism of prebiotic supplementation.

## 5. Conclusions

Using the model of ex-germ-free mice humanized with mixed human vegan microbiota we found that it does not protect against the adverse effects of a Western-type diet like obesity, liver steatosis, and compromised glucose homeostasis. In contrast, supplementation of the Western diet with inulin reversed the steatosis and ameliorated glucose metabolism, though it did not affect the weight gain. Inulin supplementation resulted in a significant change in the gut microbiota composition and its metabolic performance, inducing the shift from proteolytic towards saccharolytic fermentation. Our results offer an explanation for the relatively modest success of FMT in treating metabolic disorders when healthy microbiota were applied into an unhealthy environment without subsequent dietary support. In the context of the potential use of FMT with vegan microbiota in the therapy of metabolic non-communicable diseases, our study points out that it is not only the particular microbiota transfer, but also the following dietary intervention with inulin or other dietary fiber and/or dietary change that is necessary for therapeutic success.

## Figures and Tables

**Table 1:** Baseline clinical characteristics of stool donors. HDL-ch, HDL-cholesterol; LDL-ch, LDL-cholesterol; TC-ch, total cholesterol; TAG, serum triacylglycerol.

ID	Sex	Age	BMI	Glucose mM	TC mM	HDL-ch mM	LDL-ch mM	TAG mM	CRP Mg/L
1	F	24.5	21.8	4.86	2.8	0.89	1.33	1.28	2.1
2	M	29.1	20.3	4.65	3.23	1.6	1.45	0.41	0.3
3	M	31.2	21.4	5.04	3.68	1.21	1.93	1.2	0.3
4	F	40.5	22.5	4.9	4.58	2.16	2.12	0.66	0.5

## Data Availability

Sequencing data are available at the Sequence Read Archive database under the accession number PRJNA891477 https://www.ncbi.nlm.nih.gov/sra/PRJNA891477, accessed on 16 November 2022.

## Supplementary Materials

The following supporting information can be downloaded at: https://www.mdpi.com/article/10.3390/nu15020454/s1, Supplemental methods Table S1 The cecum microbiota composition: level L_1 (phylum). Table S2 The cecum microbiota composition: level L_6 (species). Table S3 Volatile organics compounds spectrum in the cecum. Figure S1 Alpha diversity of fecal microbiota in timepoint A assessed as Shannon index. Figure S2 The microbiota composition in timepoint A. Figure S3 Alpha diversity of cecum microbiota in timepoint B assessed as Shannon index. Figure S4 Serum metabolome composition. File S1: Supplemental Method [59,60,61,62,63,64].
