# Supplementary material for: Protective Effect of Vegan Microbiota on Liver Steatosis Is Conveyed by Dietary Fiber: Implications for Fecal Microbiota Transfer Therapy
Source: Nutrients. 2023 Jan 15;15(2):454. doi: 10.3390/nu15020454 (PMC9867259; doi:10.3390/nu15020454)
Supplement: Supplementary file 1 [file nutrients-15-00454-s001.zip › Figure S1 (diversity, timepoint A)_no_points.pdf]

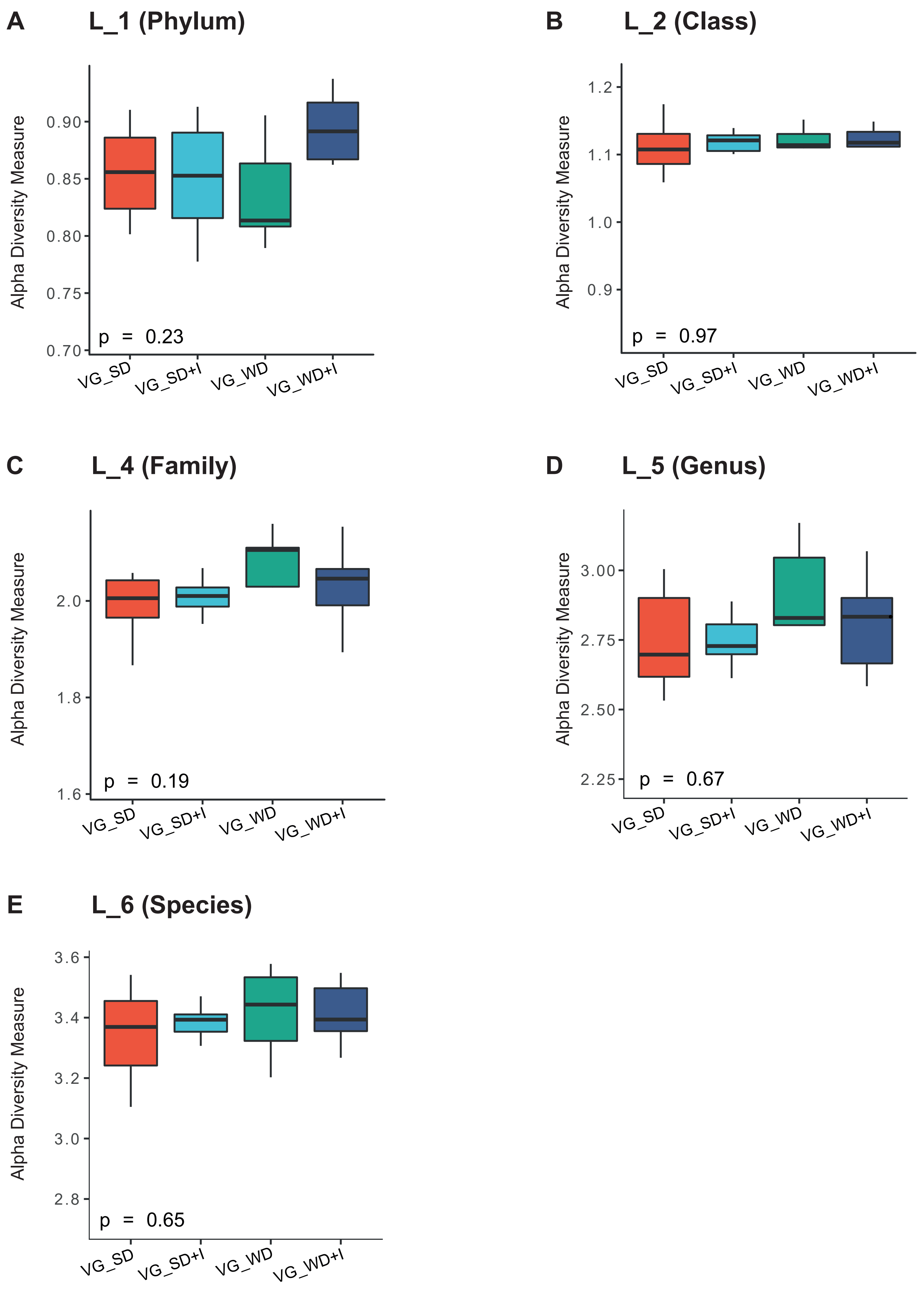

Supplementary Figure S1 *The diversity of microbiota in timepoint A.* The diversity was estimated according to the Shannon index.
