# Supplementary material for: Protective Effect of Vegan Microbiota on Liver Steatosis Is Conveyed by Dietary Fiber: Implications for Fecal Microbiota Transfer Therapy
Source: Nutrients. 2023 Jan 15;15(2):454. doi: 10.3390/nu15020454 (PMC9867259; doi:10.3390/nu15020454)
Supplement: Supplementary file 1 [file nutrients-15-00454-s001.zip › Figure S2 R1 (PCA, timepoint A).pdf]

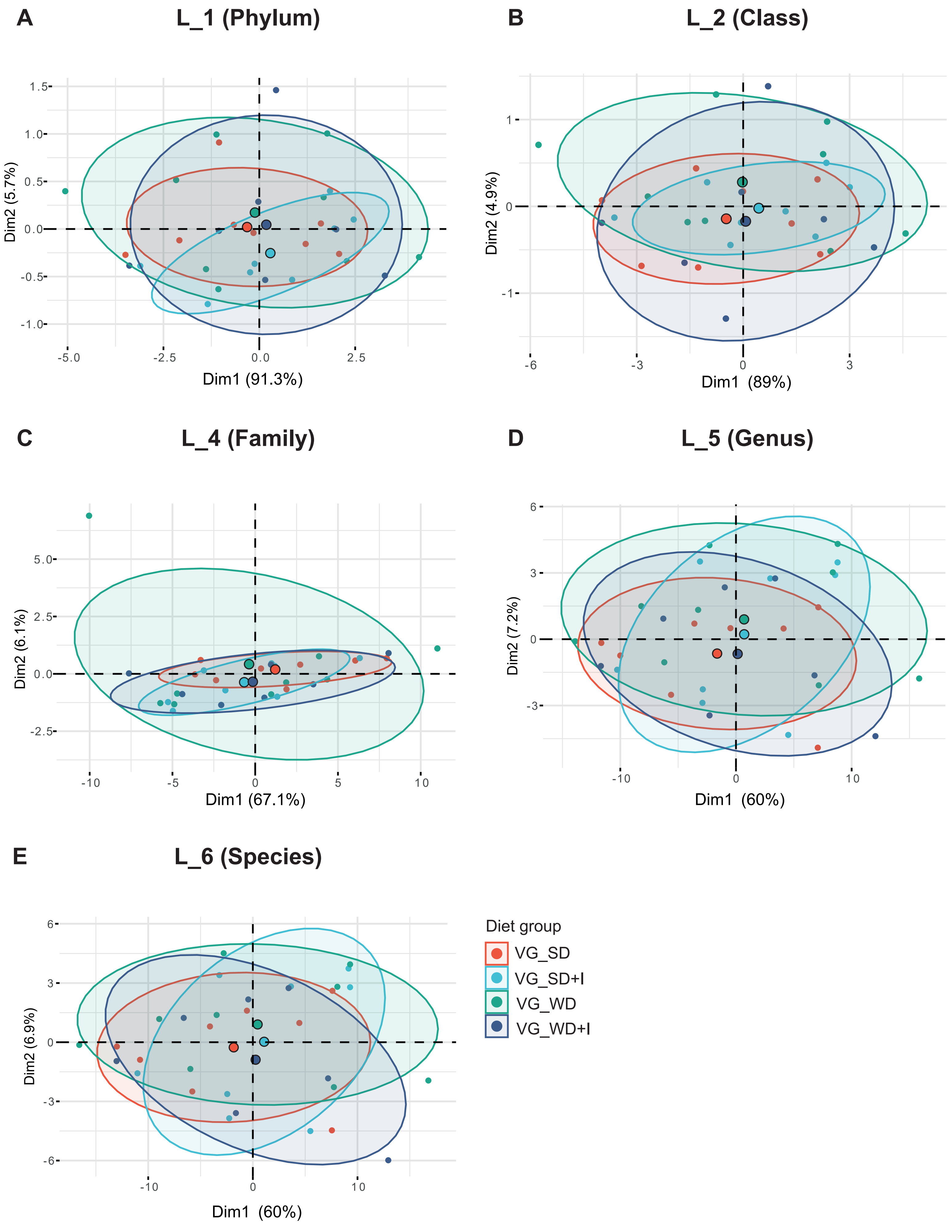

Supplementary Figure S2 *The microbiota composition in timepoint A.* The separation of the groups is visualized by PCA scores plot. The explained variance of each component is included in the axis labels. The large points represent the centroids of each group.
