# Supplementary material for: Protective Effect of Vegan Microbiota on Liver Steatosis Is Conveyed by Dietary Fiber: Implications for Fecal Microbiota Transfer Therapy
Source: Nutrients. 2023 Jan 15;15(2):454. doi: 10.3390/nu15020454 (PMC9867259; doi:10.3390/nu15020454)
Supplement: Supplementary file 1 [file nutrients-15-00454-s001.zip › Figure S3 R1 (cecum_alfa_diversity_B).pdf]

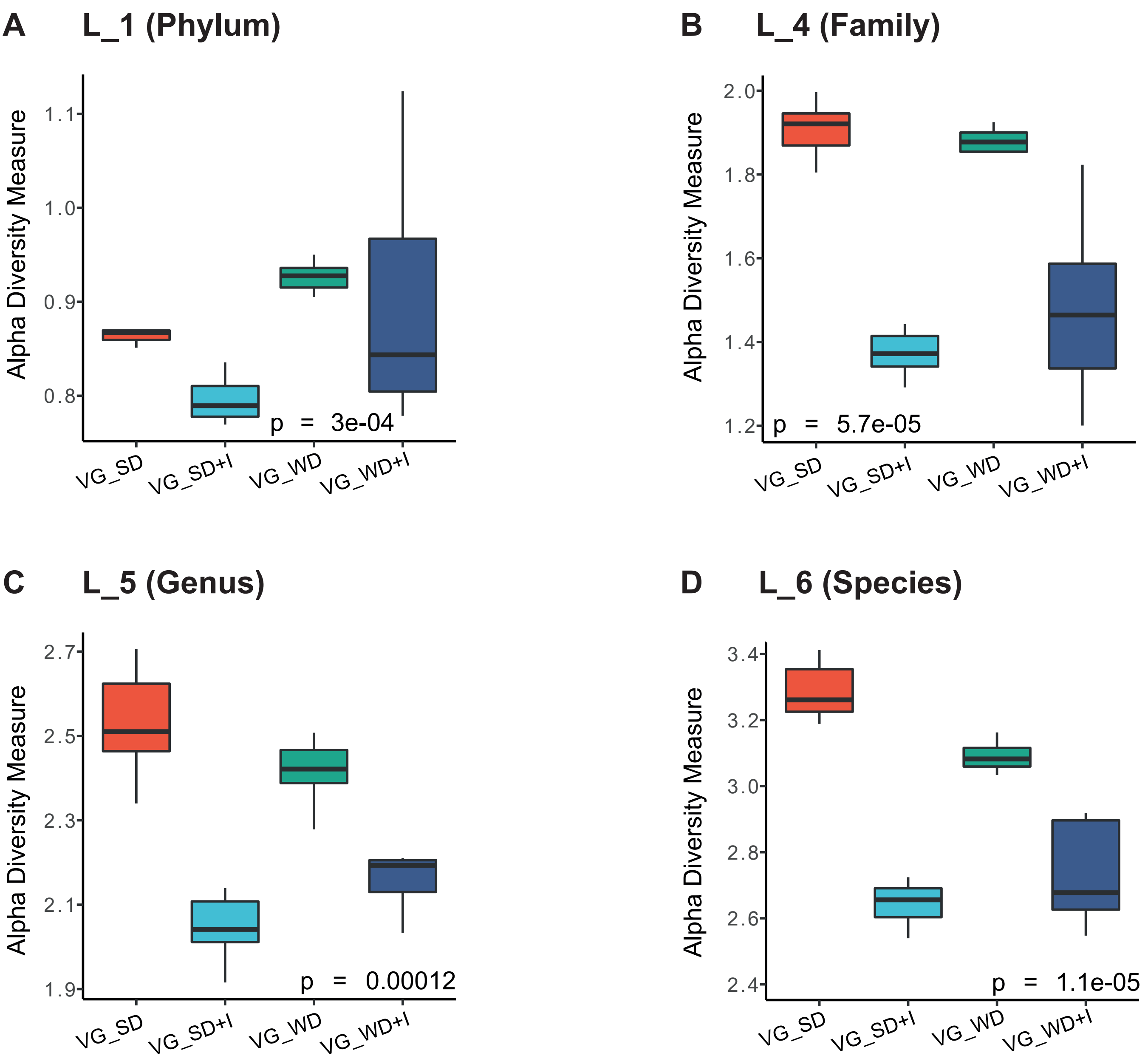

E

comparison	phylum	family	genus	species
SD to WD	0.089	0.678	0.300	0.097
SD to SD+I	0.059	0.001	0.000	0.000
SD to WD+I	0.814	0.003	0.007	0.000
WD to WD+I	0.063	0.007	0.079	0.027
SD+I to WD+I	0.074	0.646	0.269	0.649

Supplementary Figure S3 *Alpha diversity of cecum microbiota in timepoint B assessed as Shannon index.* A, taxonomic level phylum; B, taxonomic level family; C, taxonomic level Genus; D, taxonomic level Species; E, adjusted p-values.
