# Supplementary material for: Protective Effect of Vegan Microbiota on Liver Steatosis Is Conveyed by Dietary Fiber: Implications for Fecal Microbiota Transfer Therapy
Source: Nutrients. 2023 Jan 15;15(2):454. doi: 10.3390/nu15020454 (PMC9867259; doi:10.3390/nu15020454)
Supplement: Supplementary file 1 [file nutrients-15-00454-s001.zip › Figure S4 (serum).pdf]

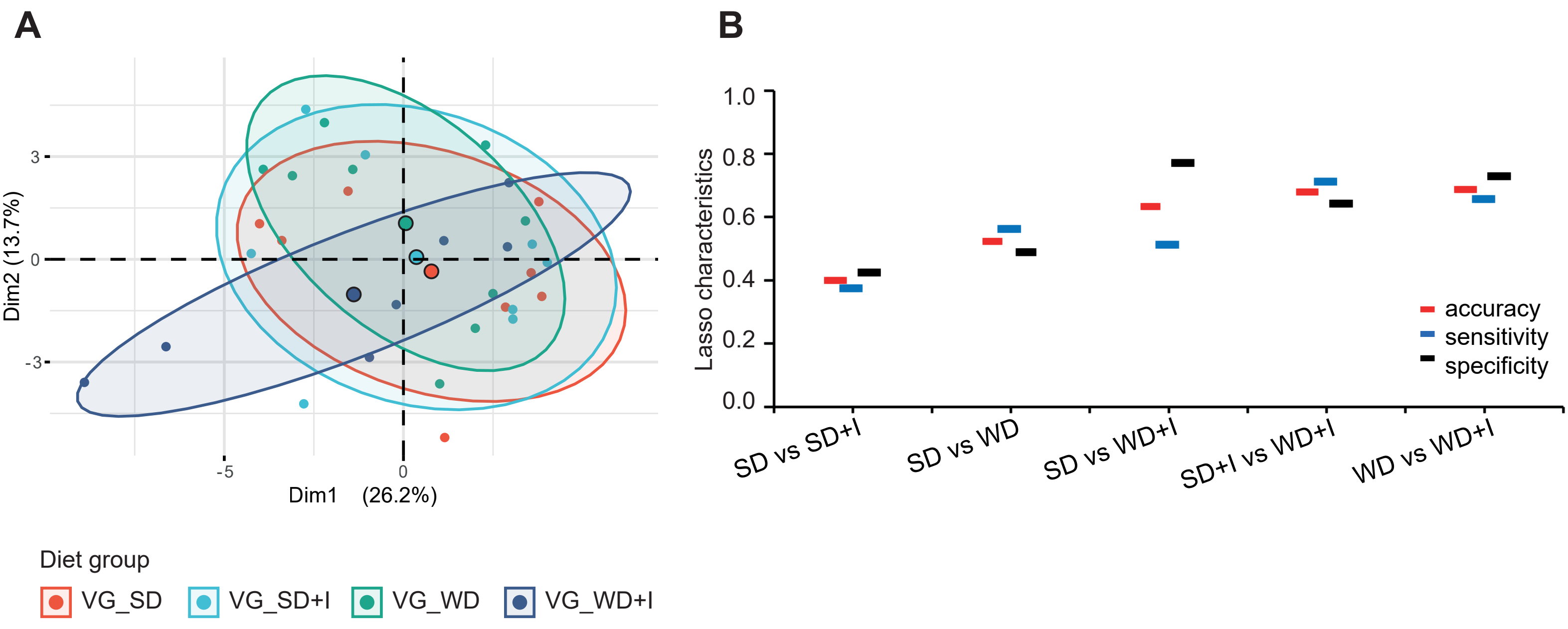

Supplementary Figure S4 *Serum metabolome composition.* A: 2D PCA scores plot. The explained variance of each component is included in the axis labels. The large points represent the centroids of each group.
