# Supplementary material for: Protective Effect of Vegan Microbiota on Liver Steatosis Is Conveyed by Dietary Fiber: Implications for Fecal Microbiota Transfer Therapy
Source: Nutrients. 2023 Jan 15;15(2):454. doi: 10.3390/nu15020454 (PMC9867259; doi:10.3390/nu15020454)
Supplement: Supplementary file 1 [file nutrients-15-00454-s001.zip › File S1.pdf]

Supplemental Methods

Donor characteristics

Four vegan donors were recruited from the vegan cohort described in detail in our previous study (Prochazkova). All of them were lean (BMI<25), healthy and they strictly avoided all animal products for at least three years**.** Their baseline clinical characteristics are given bellow.

ID	SEX	AGE	BMI	glucose mM	TC mM	HDL-ch mM	LDL-ch mM	TAG mM	transferrin g . L^{-1}
1	F	24.5	21.8	4.86	2.8	0.89	1.33	1.28	3.02
2	M	29.1	20.3	4.65	3.23	1.6	1.45	0.41	2.36
3	M	31.2	21.4	5.04	3.68	1.21	1.93	1.2	2.5
4	F	40.5	22.5	4.9	4.58	2.16	2.12	0.66	2.81

ID	ferritin µg. L^{-1}	HGB g. L^{-1}	B12 ng.mL^{-1}	folate µg. L^{-1}	PTH pM	Albumine g . L^{-1}	CRP mg .L^{-1}	bilirubin µM
1	26	120	240	7.8	5.9	46.6	2.1	11.8
2	83.6	142	184	10.1	7.1	45.9	0.3	7.9
3	54.5	143	841	7.5	4.8	52.2	0.3	8.6
4	29.6	135	500	18.1	6.8	46.2	0.5	8.7

CRP, C-reactive protein; HDL-ch, HDL cholesterol; HGB, hemoglobin; LDL-ch, LDL cholesterol; TAG, triacylglycerol; PTH, parathormone; TC, total cholesterol.

Bacteriome analysis

Sample collection and processing

Stool samples were immediately stored at -80°C until further processing. Cecum content was diluted with sterile phosphate buffered saline 1/1 (vol/vol), homogenized and aliquoted, One aliquot was used for dry mass determination, the others were stored at -80°C.

16S rRNA gene Library Preparation and Sequencing

DNA was isolated by QIAmp PowerFecal DNA Kit (Qiagen), according to manufacturer recommendation. Quality of DNA was determined using gel electrophoresis and concentration was assessed spectrophotometrically using microplate reader (Synergy Mx, BioTek, USA). For identification of bacteria presented in samples, the sequencing of 16S rRNA gene was performed. Extracted DNA was used as a template in amplicon PCR to target the hypervariable region V4 of the bacterial 16S rRNA. The library was prepared according to the Illumina 16S Metagenomic sequencing Library Preparation protocol with some deviations described below (Klindworth). The total reaction volume of PCR was 30 µL consisting of 15 µL Q5 HighFidelity 2x MM (BioLabs, New England), 1.5 µL of each 10 µM primer, 9 µL of PCR water and and 3 µL of template. The cycling parameters included initial denaturation at 98 °C for 30 s, followed by 30 cycles of 10 s denaturation at 98 °C, 15 s annealing at 55 °C and 30 s extension at 72 °C, followed by final extension at 72 °C for 2 min. The primer pair consists of Illumina overhang nucleotide sequences, an inner tag and gene-specific sequences. The Illumina overhang served to ligate the Illumina index and adapter. Each inner tag, i.e. a unique sequence of 7–9 bp, was designed to differentiate samples into groups. The amplified PCR products were determined by gel electrophoresis. PCR clean-up was performed with SPRIselect beads (Beckman Coulter Genomics). Samples with different inner tags were equimolarly pooled based on fluorometrically measured concentration using Qubit® dsDNA HS Assay Kit (Invitrogen™, USA) and microplate reader (Synergy Mx, BioTek, USA). Pools were used as a template for a second PCR with Nextera XT indexes (Illumina, USA). Differently indexed samples were checked and quantified using the three methods: qPCR using LightCycler 480 Instrument (Roche, USA) and KAPA Library Quantification Complete Kit (Roche, USA); 2100 Bioanalyzer Instrument using the High Sensitivity D1000 ScreenTape (Agilent Technologies, USA) and microplate reader (Synergy Mx, BioTek, USA) Qubit® using dsDNA HS Assay Kit (Invitrogen™, USA). Samples were equimolarly pooled according

to the measured concentration. The prepared library was checked with the same methods and concentration was measured shortly prior sequencing. The final library was diluted to a concentration of 8 pM and 20 % of PhiX DNA (Illumina, USA) was added. Sequencing was performed with the Miseq reagent kit V2 using a MiSeq instrument according to the manufacturer's instructions (Illumina, USA).

Data processing

Paired reads from 16s rRNA sequencing were first processed using an in-house pipeline implemented in Python 3. Steps of processing included trimming of low-quality 3' ends of reads, removal of read pairs containing unspecified base N and removal of pairs containing very short reads. In order to minimize sequencing and PCR-derived error, forward and reverse reads were denoised using the DADA2 amplicon denoising R package (Callahan). Following denoising, the forward and reverse reads were joined into a single longer read using the fastq-join read joining utility (Aronesty). In order to be joined, reads in pairs had to have an overlap of at least 20 base pairs with no mismatches allowed. Pairs in which this was not the case were discarded. As the final step, chimeric sequences were removed from the joined reads using the remove Bimera function of the DADA2 R package. Subsequent taxonomic assignment was conducted by the assignTaxonomy function from DADA2 R package using the Silva 138.1 reference database (McLaren).

The sequencing coverage is shown in the following table

	timepoint	group	min	Q1	median	Q3	max
Cecum							
	B	SD	15470	18680.00	20389.0	25597.00	26278
		SD + I	11027	16912.75	31425.5	43987.75	63215
		WD	4613	20931.00	29789.0	35728.00	67466
		WD + I	2010	10476.00	29037.0	37552.50	51290
Feces	A	VG / SD	4012	10988.50	17133.5	37604.00	48125
		VG / SD + I	5338	16843.25	27158.5	46006.75	61905
		VG / WD	765	12859.00	14741.0	51959.00	111594
		VG / WD + I	3503	15085.50	21382.0	34513.00	76243
	B	VG / SD	1643	9812.25	14073.5	49708.75	123371
		VG / SD + I	2668	16273.50	18623.0	21351.50	53511
		VG / WD	2690	18233.00	46511.0	48759.00	72167
		VG / WD + I	11105	14217.50	18263.0	53526.00	62961

Volatile compounds analysis of feces

Cecum content was homogenized and diluted to equivalent of 1% (*wt/wt*) dry mass. This was pippeted into a 10 mL vial for headspace analysis, and prior sealing with a magnetic cap, 20 µL of sodium azide water solution (0.2%, *wt/vol*) was added as a bacteriostatic agent. Volatiles fingerprinting was performed using an Agilent 7890B gas chromatograph coupled to Leco Pegasus 4D time of flight mass spectrometer. The instrument was equipped with a multi-purpose autosampler (MPS, Gerstel, USA), performing heated incubation, steering, and volatiles collection onto a solid-phase microextraction fiber with a divinylbenzen/carboxen/ polydimethylsiloxan (DVB/CAR/PDMS 50/30 µm) coating from Supelco (USA).

The sample was incubated for 10 min and volatiles extracted onto a fibers stationary phase for 50 minutes, both at steering at temperature of 60 °C. Separation was performed on GC capillary column DB-wax (15 m × 0.25 mm i.d., 0.25 µm film thickness; Agilent Technologies, USA) with splitless injection at 265 °C. The GC oven temperature program was as follows: 40 °C for 2 min; then ramped

at a rate of 15 °C/min to 160 °C; then at 25 °C/min to 260 °C and held for 2 min for a total GC run time of 16 min.

Time of flight mass spectrometer was operated with acquisition speed of 10 Hz to obtain full spectral information in a mass range 35–350 Da. Peak find,mass spectral deconvolution and subsequent peak alignment were performed in ChromaTOF software (LECO, USA). Compounds with a quantification mass signal to noise ratio (S/N), higher than 100 and present in more than 50 % of smallest sample class, were selected for alignment. For signals from different samples, to be listed in the aligned table as a single compound, retention time (maximal difference of 2 s) and spectral similarity at least 60 % must be met. In the aligned table, areas of quantification masses for each aligned compound, with tentative identification were provided. This tentative ID is based on spectral similarity of deconvoluted mass spectrum of signal and spectra in NIST 2017 mass spectral library. Further confirmation of signals identity was based on comparison of measured retention index and retention indexes in the NIST library. An aligned table was exported to Microsoft Excel, where constant sum normalization was performed. Thus each compounds quantification mass area was divided by sum of all signals quantification mass areas in respective sample.

NMR analysis

Serum samples were analyzed after protein precipitation. Aliquot of 220 μL serum sample was mixed with 440 μL cold methanol. The mixture was kept in freezer at -20 °C for 30 minutes and then centrifuged at 18 620 g for 10 minutes at 4 °C. The supernatant was transferred into fresh vial and vacuum dried. Evaporated supernatant was dissolved in 450 μL D_2O with 50 μL 1.5 M phosphate buffer and 50 μL 0.1% TSP, and then transferred into 5mm NMR tube.

NMR data were acquired on a 600 MHz Bruker Avance III spectrometer (Bruker BioSpin, Rheinstetten, Germany) equipped with a 5mm TCI cryogenic probe head. All experiments were performed using Topspin 3.5 software at 300 K with automatic tuning and matching, shimming and adjusting 90° pulse length for each sample. Serum data were analyzed from Carr-Purcell-Meiboom-Gill (CPMG) spectra acquired by cpmgpr1d pulse sequence with following acquisition parameters; number of scans NS=192, spectral width SW=20 ppm, 64k of data points (TD), relaxation delay for water presaturation d1=4 s, echo time 0.3 ms, loop for T2 filter 126. *J*-resolved experiment (NS=2, SW=16, TD=8k, number of increments=40, SW=78.125 Hz in the indirect dimension, d1=2 s) was performed on each sample to facilitate metabolite identification. Additional heteronuclear single quantum correlation (HSQC) and total correlation spectroscopy (TOCSY) experiments were executed for selected samples.

Acquired data were processed with Topspin 3.5 software. CPMG spectra were line broadened (0.3 Hz), automatically phased, baseline corrected and referenced to the signal of TSP. The regions with signal of water and methanol were excluded and then spectra were normalized using probabilistic quotient normalization (PQN) method (Dieterle) to the pooled lean healthy group. Individual metabolites were identified using Chenomx software (Chenomx Inc., Edmonton, AB, Canada) and their proton and carbon data were then compared with the HMDB database (Wishart). Metabolite concentrations were expressed as normalized intensities of corresponding signals in CPMG spectra.

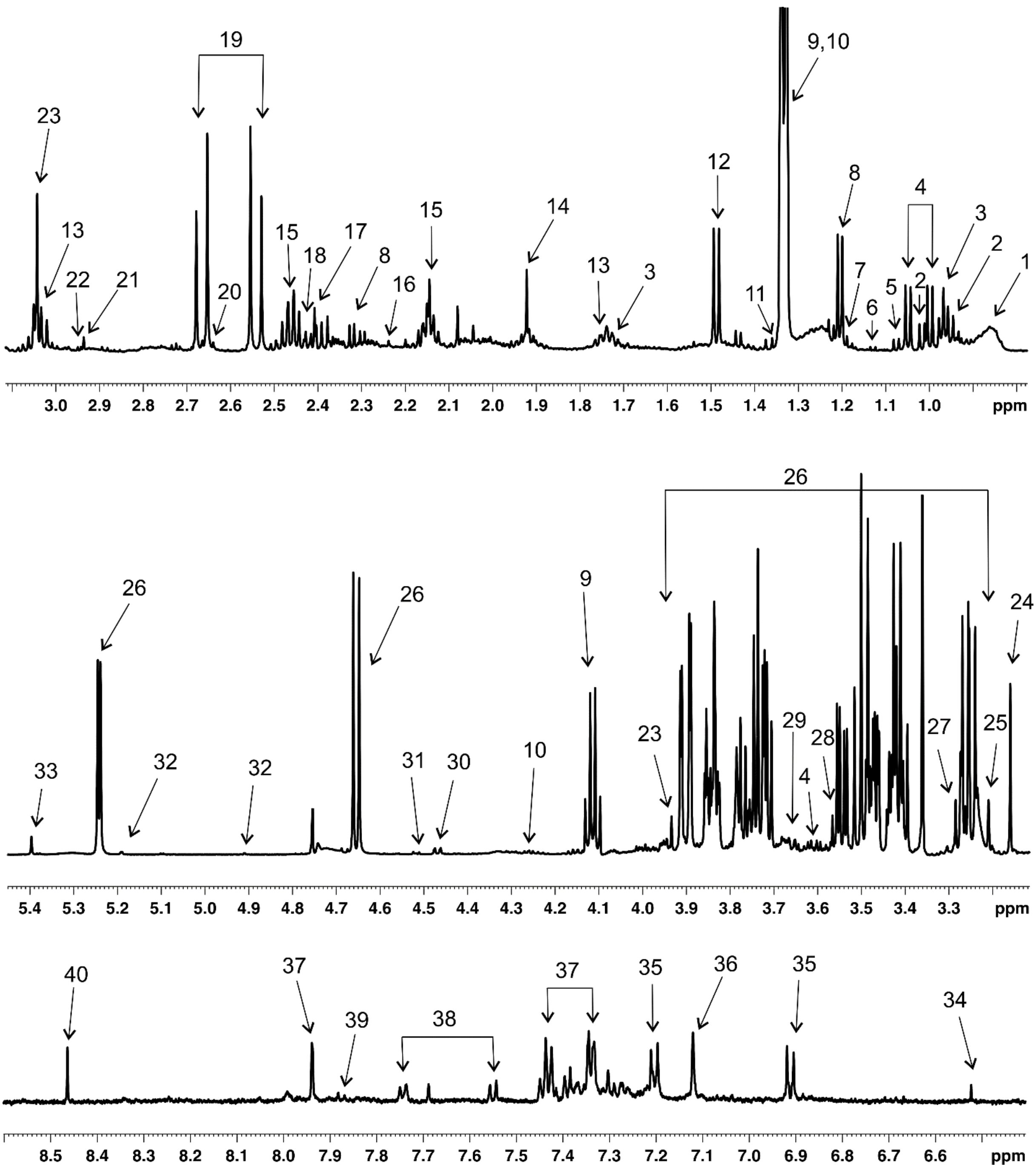

*Repesentative ^{1}H NMR spectrum of serum with quantified metabolites*1: lipids; 2: isoleucine; 3: leucine; 4: valine; 5: 3-hydroxyisobutyrate; 6: 2-oxoisovalerate; 7: ethanol; 8: 3-hydroxybutyrate; 9: lactate; 10: threonine; 11: 2-hydroxyisobutyrate; 12: alanine; 13: lysine; 14: acetate; 15: glutamine; 16: acetone; 17: pyruvate; 18: succinate; 19: citrate; 20: methionine; 21: dimethylglycine; 22: asparagine; 23: creatine; 24: malonate; 25: choline; 26: glucose; 27: taurine; 28: glycine; 29: glycerol; 30: lactate; 31: ascorbate; 32: mannose; 33: allantoin; 34: fumarate; 35: tyrosine; 36: histidine; 37: phenylalanine; 38: tryptophan; 39: uridine; 40: formate

Histological evaluation of liver tissue sections

Liver tissue was fixed overnight in 4% paraformaldehyde (pH 7.4) at 4 °C and routinely processed for histological examination. Sections cut at 4-6 µm were stained with hematoxylin and eosin and examined with an Olympus BX41 light microscope.

All samples were semiquantitatively evaluated for the presence of steatosis, inflammation, and ballooning degeneration according to Kleiner et al. Steatosis was graded as “0” (<5% liver parenchyma involved by steatosis), “1” (5%-33 %), “2” (>33%-66%) and “3” (>66%). The prevalent type of hepatic steatosis (microvesicular, macrovesicular, or mixed) was determined.

Supplemental references

Aronesty,E. Comparison of sequencing utility programs. Open Bioinform. J., 7, 1–8 (2013).

Callahan B.J., McMurdie P.J., Rosen M.J., Han A.W., Johnson A.J.A., Holmes S.P. DADA2: High-resolution sample inference from Illumina amplicon data, Nat Methods 13, 581–583 (2016).

Caporaso J., Kuczynski J., Stombaugh J., Bittinger K., ET AL. QIIME allows analysis of high-throughput community sequencing data, Nat Methods 7, 335–336 (2010).

Dieterle F., Ross A., Schlotterbeck G., Senn H., Probabilistic quotient normalization as robust method to account for dilution of complex biological mixtures. Application in 1H NMR metabonomics. Anal Chem. 78(13), 4281-4290 (2006)

Kleiner DE, Brunt EM, Van Natta M, Behling C, Contos MJ, Cummings OW, Ferrell LD, Liu YC, Torbenson MS, Unalp-Arida A, Yeh M, McCullough AJ, Sanyal AJ; Nonalcoholic Steatohepatitis Clinical Research Network. Design and validation of a histological scoring system for nonalcoholic fatty liver disease. Hepatology. 2005 Jun;41(6):1313-21. doi: 10.1002/hep.20701.

Klindworth, A. *et al*. Evaluation of general 16S ribosomal RNA gene PCR primers for classical and next-generation sequencing-based diversity studies. Nucleic Acids Res. 41, e1 (2013).

Quast, C. et al. The SILVA ribosomal RNA gene database project: improved data processing and web-based tools. Nucleic Acids Res. 41, D590–D596 (2013). McLaren, Michael R., & Callahan, Benjamin J. (2021). Silva 138.1 prokaryotic SSU taxonomic training data formatted for DADA2 [Data set]. Zenodo. https://doi.org/10.5281/zenodo.4587955

Prochazkova M, Budinska E, Kuzma M, Pelantova H, Hradecky J, Heczkova M, et al. Vegan Diet Is Associated With Favorable Effects on the Metabolic Performance of Intestinal Microbiota: A Cross-Sectional Multi-Omics Study. Front Nutr. 2021;8:783302.

Wishart D.S. *et al*. HMDB 4.0: the human metabolome database for 2018. Nucleic Acids Res. 46(D1), D608-D617 (2018)
